# Small molecular modulation of macrophage migration inhibitory factor in the hyperoxia-induced mouse model of bronchopulmonary dysplasia

**DOI:** 10.1186/1465-9921-14-27

**Published:** 2013-02-28

**Authors:** Huanxing Sun, Rayman Choo-Wing, Juan Fan, Lin Leng, Mansoor A Syed, Alissa A Hare, William L Jorgensen, Richard Bucala, Vineet Bhandari

**Affiliations:** 1 Department of Pediatrics, Yale University, New Haven, CT, 06520, USA; 2 Department of Medicine, Yale University, New Haven, CT, 06520, USA; 3 Department of Chemistry, Yale University, New Haven, CT, 06520, USA; 4 Yale Child Health Research Center, Yale University School of Medicine, 464 Congress Avenue, P.O. Box 208081, New Haven, CT, 06520-8081, USA

**Keywords:** Newborn, Oxygen, Bronchopulmonary dysplasia

## Abstract

**Background:**

The role and mechanism of action of MIF in bronchopulmonary dysplasia (BPD) are not known. We hypothesized that increased MIF signaling would ameliorate the pulmonary phenotype of BPD in the mouse lung.

**Methods:**

We studied newborn wild type (WT), MIF knockout (MIFKO), and lung MIF transgenic (MIFTG) mice in room air and a BPD model, and examined the effects of administering a small molecule MIF agonist and antagonist. Lung morphometry was performed and mRNA and protein expression of vascular mediators were analyzed.

**Results:**

The pulmonary phenotype of MIFKO and MIFTG mice lungs in room air (RA) and BPD model were comparable to the WT-BPD mice at postnatal (PN) day 14. Vascular endothelial growth factor (VEGF)-A, -R1 and Angiopoietin (Ang)1 mRNA were decreased, and Ang2 increased in the WT-BPD, MIFKO-RA, MIFKO-BPD, MIFTG-RA and MIFTG-BPD mice lungs, compared to appropriate controls. The protein expression of Ang1 in the MIFKO-RA was similar to WT-RA, but decreased in MIFTG-RA, and decreased in all the BPD groups. Ang2 was increased in MIFKO-RA, MIFTG-RA and in all 3 BPD groups. Tie2 was increased in WT-BPD compared to WT-RA, but decreased in MIFKO- and MIFTG- RA and BPD groups. VEGFR1 was uniformly decreased in MIFKO-RA, MIFTG-RA and in all 3 BPD groups. VEGF-A had a similar expression across all RA and BPD groups. There was partial recovery of the pulmonary phenotype in the WT-BPD model treated with the MIF agonist, and in the MIFTG mice treated with the MIF antagonist.

**Conclusions:**

These data point to the careful regulatory balance exerted by MIF in the developing lung and response to hyperoxia and support the potential therapeutic value of small molecule MIF modulation in BPD.

## Background

The pathogenesis of bronchopulmonary dysplasia (BPD), which is the most common chronic lung disease of infancy, is multifactorial, with important contributions from both genetic and environmental factors [1]. The pulmonary phenotype of BPD is that of impaired alveolarization, as exemplified by large, simplified alveoli and dysregulated vascularization [1]. The anatomic foundation of BPD is that of lung immaturity. We previously reported that fetal murine lungs deficient in the growth regulatory and inflammatory cytokine, macrophage migration inhibitory factor (MIF), show impaired lung maturation [2]. In addition, human infants who have adverse pulmonary outcome or develop BPD have been reported to have lower tracheal aspirate levels of MIF [2,3] and are more likely to have a low expression *MIF* allele [4]. The specific role and mechanism of action of MIF in BPD are not known.

Exposure of the developing lung to hyperoxia is a critical factor in the occurrence of BPD [1,5], thus highlighting the need for understanding the role of hyperoxia among the environmental factors contributing to “new” BPD [1,6,7]. An improved understanding of the mechanisms of hyperoxia-induced lung injury in the context of the role of host mediators such as MIF would be helpful in formulating potential therapeutic strategies with the goal of ameliorating BPD [7,8].

We hypothesized that increased MIF signaling would ameliorate the pulmonary phenotype of BPD in the mouse lung. To address this hypothesis, we studied newborn (NB) mice genetically deficient in MIF (MIF knock out: MIFKO). We also examined the phenotypic response of novel lung-targeted MIF overexpressing transgenic (MIFTG) mice. These genetically defined mouse strains were further subjected to a mouse model of BPD, sacrificed at post natal (PN) day 14. In addition, we studied the effects of administering novel, small molecule modulators of MIF signal transduction [9,10].

Our goal was to study alterations in pulmonary phenotype and the expression of vascular mediators in the varied mouse models. Specifically, we evaluated lung morphometry and the expression of vascular endothelial growth factor (VEGF), its receptors (R1-3), angiopoietin (Ang), and its receptor (Tie2) in the lung.

## Methods

### Animals

MIFKO mice were backcrossed for this study into the C57BL/6 genetic background (generation N10) [2]. We have recently reported the generation and characterization of the pulmonary phenotype of the lung-targeted MIF transgenic (MIFTG) mouse [11]. Briefly, we targeted the lung by using rat Clara Cell 10kD protein (CC10), which is a Type II epithelial-cell and conducting airway-cell specific promoter in the developing lung [12]. We developed the TG mice on the C57BL/6 background, and confirmed the constitutive MIF mRNA and protein overexpression in lung tissue and bronchoalveolar lavage (BAL) fluid.

All animal work was approved by the Institutional Animal Care and Use Committee at the Yale University School of Medicine.

### Newborn mouse BPD model

For the NB animals, exposure to hyperoxia (along with their mothers) was performed by placing mice in cages in an airtight Plexiglass chamber (55 × 40 × 50 cm), as described previously [13,14]. For the NB mouse model of BPD, exposure to 100% oxygen was initiated on PN1 and continued until PN4 (saccular stage of mouse lung development) and allowed to recover in room air (RA) for the next 10 days (alveolar stage of mouse lung development). Two lactating dams were used and alternated in hyperoxia and RA every 24 h, during the hyperoxia phase (PN1-4) of the experimental model. The litter size was limited to 10–12 pups per dam to control for the effects of litter size on nutrition and growth. Mice were sacrificed on PN14. Using this experimental model, NB WT mouse lungs at PN14 have the phenotype mimicking human BPD, as has been reported previously by us [15] and other investigators [16].

Throughout the experiment, mice were given free access to food and water, and oxygen levels were continually monitored. The inside of the chamber was maintained at atmospheric pressure and mice were exposed to a 12 hr light–dark cycle. We opened the oxygen chamber once a day (from PN1-4; they were in RA from PN5-14) to change the mothers and inject the mouse pups with the MIF agonist and antagonist molecules [9]. The MIF agonist, 4-(4-(pyridin-3-yl)-1H-1,2,3-triazol-1-yl) phenol, designated MIF020 (compound 5a) [9] and the MIF antagonist, 3-(3-hydroxybenzyl)-5-methylbenzooxazol-2-one, designated MIF098 (compound 5) [10] were dissolved in 10% DMSO (Vehicle 1) at a concentration of 0.04 mg/μl, and administered intra-dermally from PN1-4 and intra-peritoneally from PN5-14, at a dose of 0.4 mg/g/day.

### Analysis of mRNA

RNA was isolated from frozen lungs using TRIzol Reagent (Invitrogen Corporation, Carlsbad, CA) and treated by DNase. RNA samples were then purified by RNeasy kit (Qiagen Sciences, Maryland, USA) according to the manufacturer’s instructions. RNA samples were subjected to semi-quantitative RT-PCR.

The primers used for semi-quantitative RT-PCR are as follows:

β-Actin-F, 5’-GTGGGCCGCTCTAGGCACCA -3’

β-Actin-R, 5’-TGGCCTTAGGGTTCAGGGGG -3’

MIF-F, 5’- ATGCCTATGTTCATCGTGA -3’

MIF-R, 5’-TCAAGCGAAGGTGGAACCGTT −3

VEGF-A-F, 5’- GACCCTGGCTTTACTGCTGTA -3’

VEGF-A-R, 5’- GTGAGGTTTGATCCGCATGAT -3’

VEGF-C-F, 5’- AACGTGTCCAAGAAATCAGCC -3’

VEGF-C-R, 5’- AGTCCTCTCCCGCAGTAATCC -3’

VEGF-R1-F, 5- CACCACAATCACTCCAAAGAAA -3’

VEGF-R1-R, 5- CACCAATGTGCTAACCGTCTTA -3’

VEGF-R2-F, 5- ATTGTAAACCGGGATGTGAAAC -3’

VEGF-R2-R, 5- TACTTCACAGGGATTCGGACTT -3’

VEGF-R3-F, 5- GCTGTTGGTTGGAGAGAAGC -3’

VEGF-R3-R, 5- TGCTGGAGAGTTCTGTGTGG -3’

Ang1-F, 5’- AGGCTTGGTTTCTCGTCAGA -3’

Ang1-R, 5’- TCTGCACAGTCTCGAAATGG -3’

Ang2-F, 5’- GAACCAGACAGCAGCACAAA -3’

Ang2-R, 5’- AGTTGGGGAAGGTCAGTGTG -3’

Tie2-F, 5’- GGACAGTGCTCCAACCAAAT -3’

Tie2-R, 5’- TTGGCAGGAGACTGAGACCT -3’

mRNA band densities were measured by densitometry using NIH image J and expressed in Arbitrary Densitometric Units (ADU), as previously described [12].

### Histology

Lung tissues obtained from the NB mice from the RA and BPD model experiments at PN14 were subjected to a standard protocol for lung inflation (25 cm) and fixed overnight in 10% buffered formalin [17]. After washing in fresh PBS, fixed tissues were dehydrated, cleared, and embedded in paraffin by routine methods. Sections (5 μm) were collected on Superfrost Plus positively charged microscope slides (Fisher Scientific Co., Houston, Texas, USA), deparaffinized, and stained with hematoxylin & eosin, as described previously [14].

### Lung morphometry

Alveolar size was estimated from the mean chord length of the airspace, as described previously [14]. Chord length increases with alveolar enlargement.

### Bronchoalveolar lavage (BAL) fluid total cell counts

BAL fluid was obtained and total cell counts enumerated, as previously reported [18].

### Western blotting

We detected MIF, Ang1, Ang2, Tie2, VEGF-A, and VEGFR1 protein with β-actin as control from lung lysates using Western analysis, as described previously [14,19]. Anti-MIF specific and Tie2 antibodies were purchased from Abcam (Cambridge, MA). Ang1 and Ang2 antibodies were purchased from Millipore (Billerica, MA). VEGF-A and β-actin antibodies were purchased from Santa Cruz Biotechnology, Santa Cruz, CA. VEGFR1 antibody was obtained from Sigma-Aldrich, St. Louis, MO.

### Statistical analyses

Values are expressed as mean ± SEM. Groups were compared with the Student's two-tailed unpaired *t* test, using GraphPad Prism 3.0 (GraphPad Software, Inc., San Diego, CA), as appropriate. A *P<*0.05 was considered statistically significant.

## Results

### Effect of hyperoxia on MIF expression in NB WT mice lungs and the mouse BPD model

**Figure 1:**
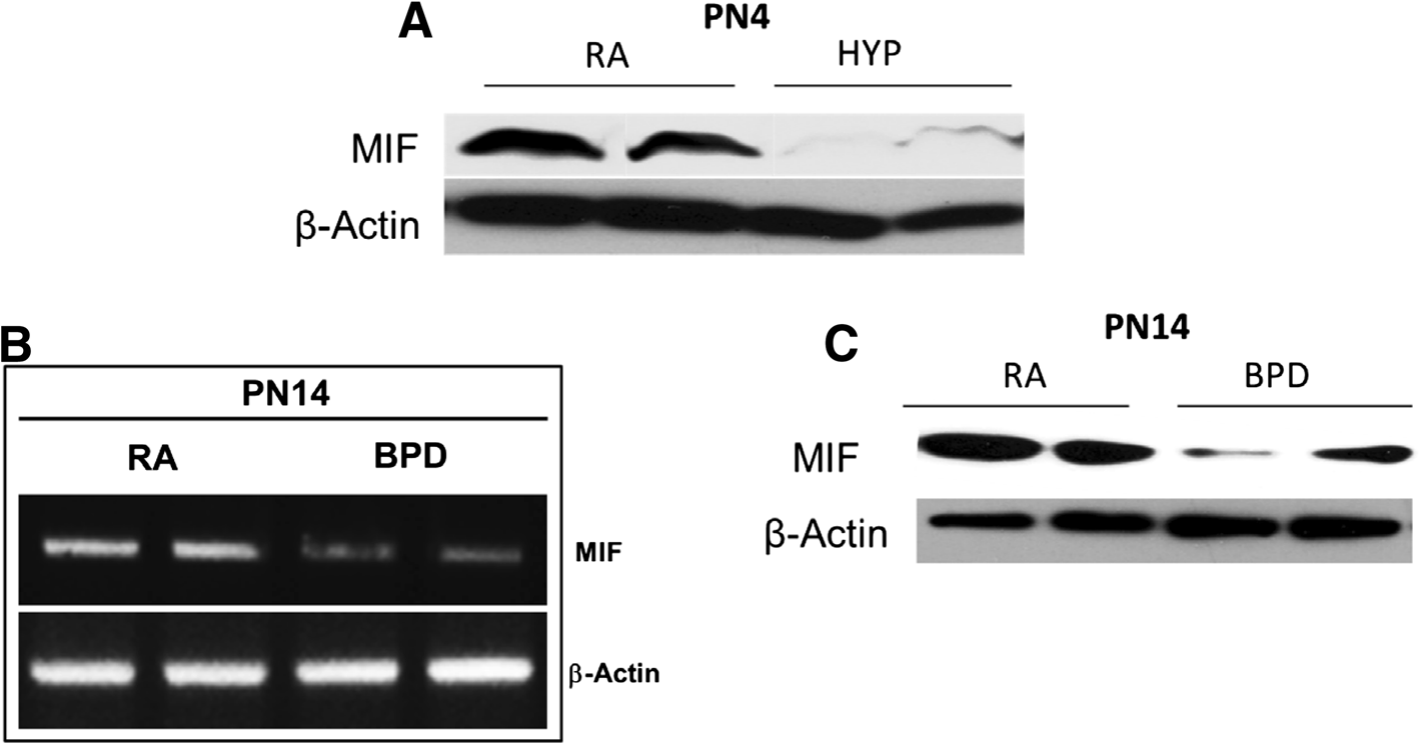
**Effect of hyperoxia on MIF expression in NB WT mice lungs and the mouse BPD model.** (**A**) Western blot of MIF protein expression in lungs of NB mice exposed to hyperoxia (as described in “Methods” section) from PN1 to PN4. Control lungs were exposed to RA for 4 PN days. The figure is representative of n=3-4 mice per group. (**B**) Semi-quantitative RT-PCR of MIF mRNA expression in lungs of NB BPD mouse model and control mice exposed to RA, at PN14. (**C**) Western blot of MIF protein expression in lungs of NB BPD mouse model and control mice exposed to RA, at PN14. The mouse BPD model was generated (described in “Methods” section), as previously reported [15]. Control mice were exposed to RA for 14 days. The figure is representative of n=3-4 mice per group. β: beta; HYP: hyperoxia; MIF: macrophage migration inhibitory factor; PN: post natal; RA: room air; BPD: bronchopulmonary dysplasia mouse model.

In independent experiments, using the NB mouse, we noted significantly decreased expression of MIF protein (Figure 1A) in WT mice lungs exposed to 100% O_2_ at PN4. At PN14, using the NB BPD model, we noted some recovery of MIF mRNA (Figure 1B) and protein (Figure 1C) expression, but MIF expression was still markedly decreased compared to RA controls.

Taken together these data suggest that exposure to hyperoxia during the critical stage of saccular phase of lung development (PN1-4) leads to decreased MIF levels in the lung, which persists even after recovery in RA for the next 10 days (PN5-14).

### Role of MIF in the mouse BPD model: impact on pulmonary phenotype and BAL total cell counts

We [15] and others [16,20] have reported on a mouse model of BPD created by exposure of WT mice to hyperoxia during the saccular stage of lung development (PN1-4), followed by RA recovery for 10 days. This model has clinical significance to human BPD in pulmonary phenotype, and long-term physiologic consequences [5,21].

**Figure 2:**
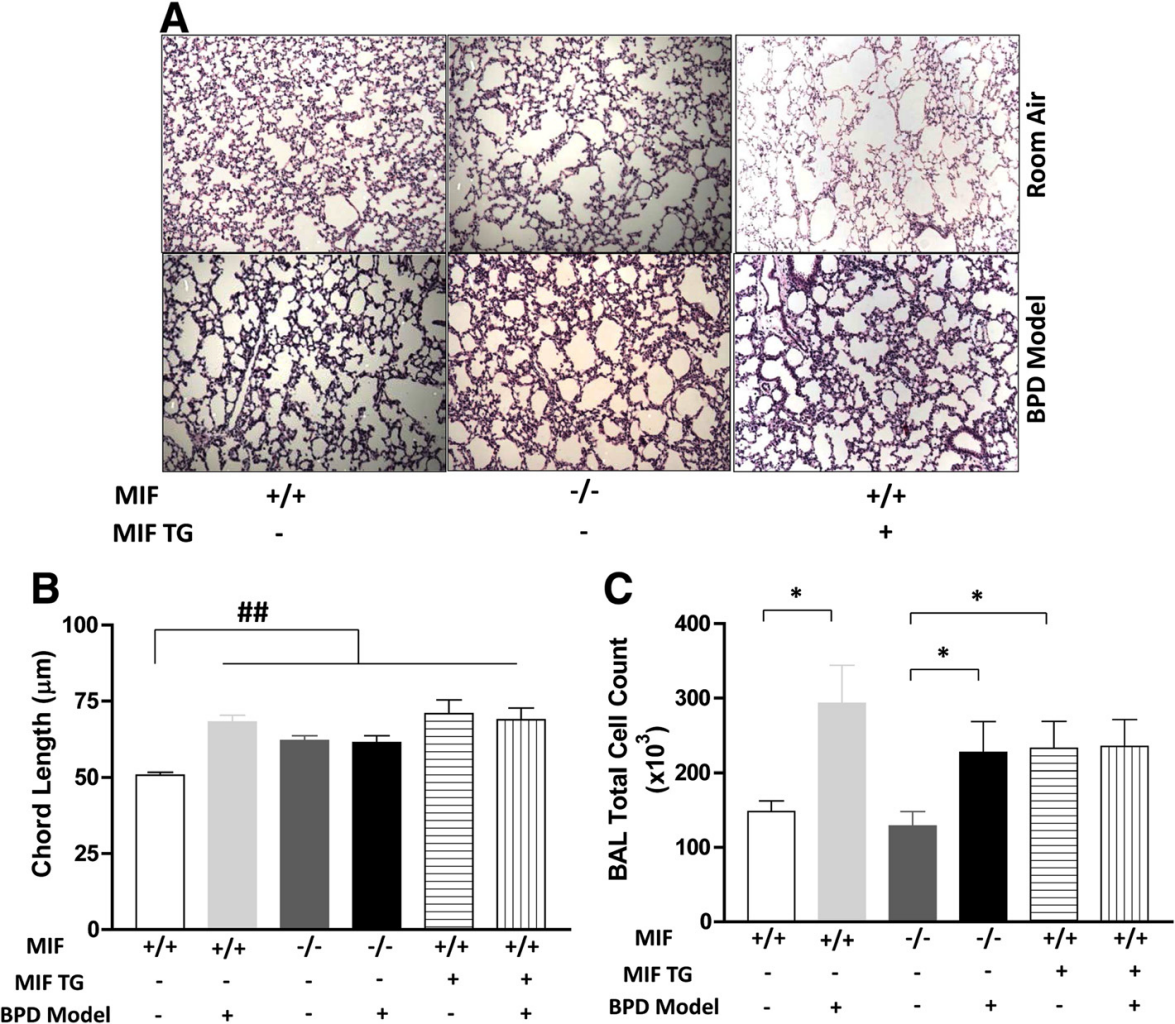
**Role of MIF in the mouse BPD model: impact on pulmonary phenotype and BAL total cell counts.** NB MIF KO, MIF TG and WT control mice were exposed to 100% O_2_ for PN1-4, with recovery in RA from PN5-14 (BPD model). Representative photomicrographs of lung histology (H&E stain) of NB MIF KO, MIF TG and WT control mice exposed to RA or BPD model at PN14 (**2A**). The figures are illustrative of a minimum of 7 animals in each group. Alveolar size, as measured by chord length, confirmed features noted on lung histology (**2B**). Each bar represents the mean ± SEM of a minimum of 7 animals. BAL total cell count of NB MIF KO, MIF TG and WT control mice exposed to RA at PN14 or BPD model (**2C**). Each bar represents the mean ± SEM of a minimum of 5 animals. MIF +/+: wild type; MIF −/−: macrophage migration inhibitory factor knock out; MIF TG +: macrophage migration inhibitory factor (over-expressing) transgenic; BAL: bronchoalveolar lavage; BPD model: bronchopulmonary dysplasia mouse model. *P<0.05; ##*P*<0.0001.

MIFKO and MIFTG mice in RA had lungs with altered alveolar architecture characterized by alveolar simplification at PN14 when compared to WT mice in RA (Figure 2A). As expected, WT mice subjected to hyperoxia from PN1-4, followed by recovery for next 10 days showed alveolar simplification characteristic of BPD (Figure 2A). However, the exposure of MIFKO and MIFTG mouse pups to hyperoxia from PN1-4 followed by recovery for next 10 days did not further worsen the pulmonary phenotype than that observed in RA, and was confirmed by lung morphometry (Figure 2B). The total BAL fluid cell counts were increased in the WT and MIFKO mice in the BPD model at PN14 when compared to respective controls (Figure 2C). Interestingly, the MIFTG mice in RA and the BPD model had similarly high values of the total BAL fluid cell counts (Figure 2C).

These data indicate that both, a lack or an excess of MIF in the developing lung leads to significant alterations in pulmonary architecture. This result highlights the likely requirement for “just the right amount” of MIF for normal lung development (“Goldilocks effect”). In addition, it suggests that excess MIF concentrations in RA in MIFTG mice appear to have an equivalent impact on pulmonary inflammation (based on total BAL cell counts) and phenotype as in the BPD model. MIF’s impact in the developing lung is thus similar to the effect of hyperoxia, with the latter having presumptive effects on a potential number of molecular mediators.

### Role of MIF in the mouse BPD model: impact on mRNA and protein expression of vascular factors and their receptors

**Figure 3:**
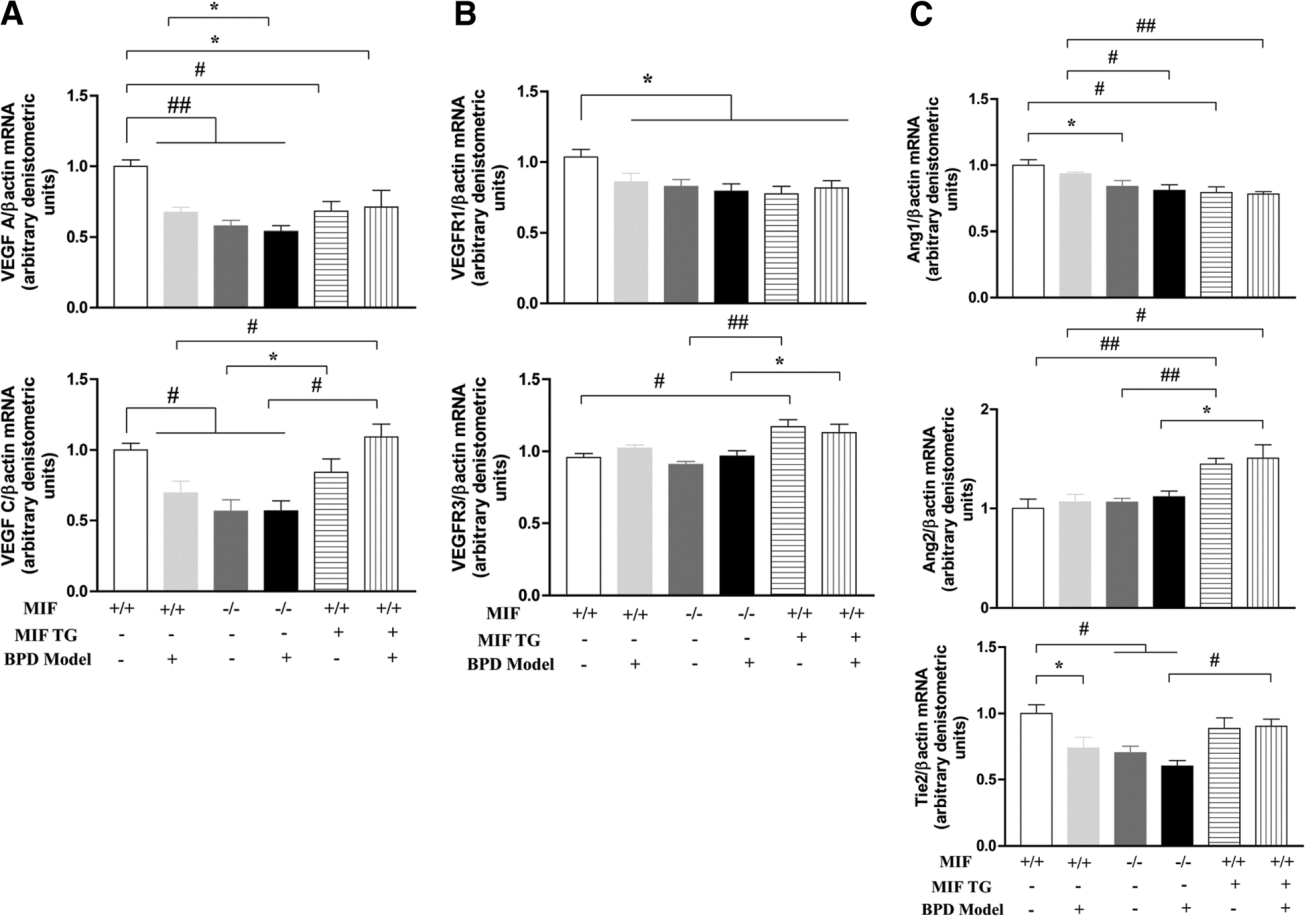
**Role of MIF in the mouse BPD model: impact on mRNA expression of vascular factors and their receptors.** NB MIF KO, MIF TG and WT control mice were exposed to 100% O_2_ for PN1-4, with recovery in RA from PN5-14 (BPD model). The mice were sacrificed at PN14. The ratios of mRNA of VEGF -A, -C and their receptors -R1 and -R3 (**3A** and **3B**) as well as Ang1 and Ang2 and their receptor Tie2 (**3C**) with β-actin were quantified by densitometry. The noted values represent assessments in a minimum of 7 animals in each group. The figure is representative of n=3-4 mice per group. MIF +/+: wild type; MIF −/−: macrophage migration inhibitory factor knock out; MIF TG +: macrophage migration inhibitory factor (over-expressing) transgenic; BPD: bronchopulmonary dysplasia mouse model. VEGF: vascular endothelial growth factor; R: receptor; Ang: angiopoietin; β-actin: beta-actin. ##*P*<0.0001, #*P*≤0.01, *P<0.05.

VEGF-A mRNA expression was uniformly decreased in the WT-BPD, MIF KO-RA, MIF KO-BPD, MIF TG-RA and MIF TG-BPD mouse lungs when compared to WT-RA control mice lungs (Figure 3A). Interestingly, VEGF-A expression was significantly decreased in the MIF KO-BPD lung compared to the WT-BPD control lungs (Figure 3A), which is consistent with the association of decreased VEGF in animal models and humans with BPD [14].

VEGF-R1 mRNA expression was uniformly decreased in the WT-BPD, MIF KO-RA, MIF KO-BPD, MIF TG-RA and MIF TG-BPD mouse lungs when compared to WT-RA control mouse lungs (Figure 3B). No differences were noted in VEGF-R2 mRNA expression among the groups (data not shown). With respect to Ang, the expression of Ang1 mRNA expression was significantly decreased in MIF KO-RA and MIF TG-RA mouse lungs when compared with WT-RA mouse lungs (Figure 3C). A similar decrease was noted in the MIF KO-BPD and MIF TG-BPD mice when compared to its control, WT-BPD (Figure 3C). In contrast, Ang2 mRNA expression was significantly increased in the MIF TG-RA and MIF TG-BPD lungs when compared to WT-RA and MIF KO-RA and WT-BPD and MIF KO-BPD lungs, respectively (Figure 3C).

The receptor for Ang 1 and 2 is Tie2. The expression of Tie2 was significantly decreased in the WT-BPD, MIF KO-RA, and MIF KO-BPD lungs when compared to WT-RA lungs (Figure 3C). By contrast, the expression of Tie2 was increased in the MIF TG-BPD lungs when compared with the MIF KO-BPD lungs (Figure 3C).

**Figure 4:**
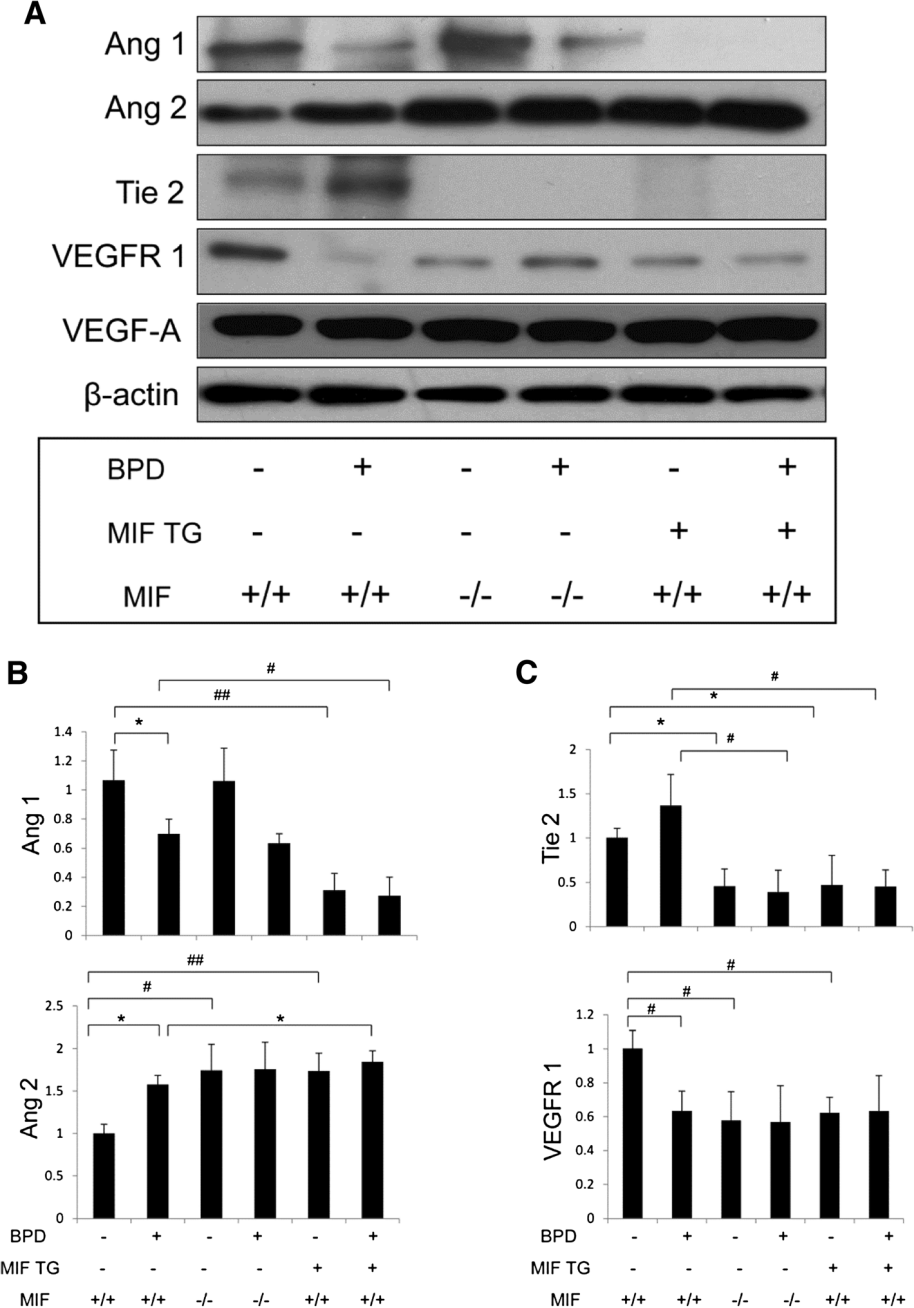
**Role of MIF in the mouse BPD model: impact on protein expression of vascular factors and their receptors.** NB MIF KO, MIF TG and WT control mice were exposed to 100% O_2_ for PN1-4, with recovery in RA from PN5-14 (BPD model). The mice were sacrificed at PN14. Ang1, Ang2, Tie2, VEGF-A, and VEGFR1 proteins, with β-actin as controls, were detected by western blotting (**4A**). The relative density of VEGF -A and it’s receptor -R1 as well as Ang1 and Ang2 and their receptor Tie2 (**4B** and **4C**) were quantified by densitometry. The figure is representative of n=3 mice per group. MIF +/+: wild type; MIF −/−: macrophage migration inhibitory factor knock out; MIF TG +: macrophage migration inhibitory factor (over-expressing) transgenic; BPD: bronchopulmonary dysplasia mouse model. VEGF: vascular endothelial growth factor; R: receptor; Ang: angiopoietin; β-actin: beta-actin. ##*P*<0.001, #*P*≤0.01, *P<0.05.

We confirmed the RNA data at the protein level (Figures 4A-C). The protein expression of Ang1 in the MIFKO-RA was similar to WT-RA, but decreased in MIFTG-RA, and decreased in all the BPD groups. Ang2 was increased in MIFKO-RA, MIFTG-RA and in all 3 BPD groups. Tie2 was increased in WT-BPD compared to WT-RA, but decreased in MIFKO- and MIFTG- RA and BPD groups. VEGFR1 was uniformly decreased in MIFKO-RA, MIFTG-RA and in all 3 BPD groups. VEGF-A had a similar expression across all RA and BPD groups. Taken together, these data would suggest an important role of the Ang-Tie2 axis in relation to MIF signaling in the mouse BPD model.

### Role of MIF agonist in the mouse BPD model: impact on pulmonary phenotype and BAL total cell counts

**Figure 5:**
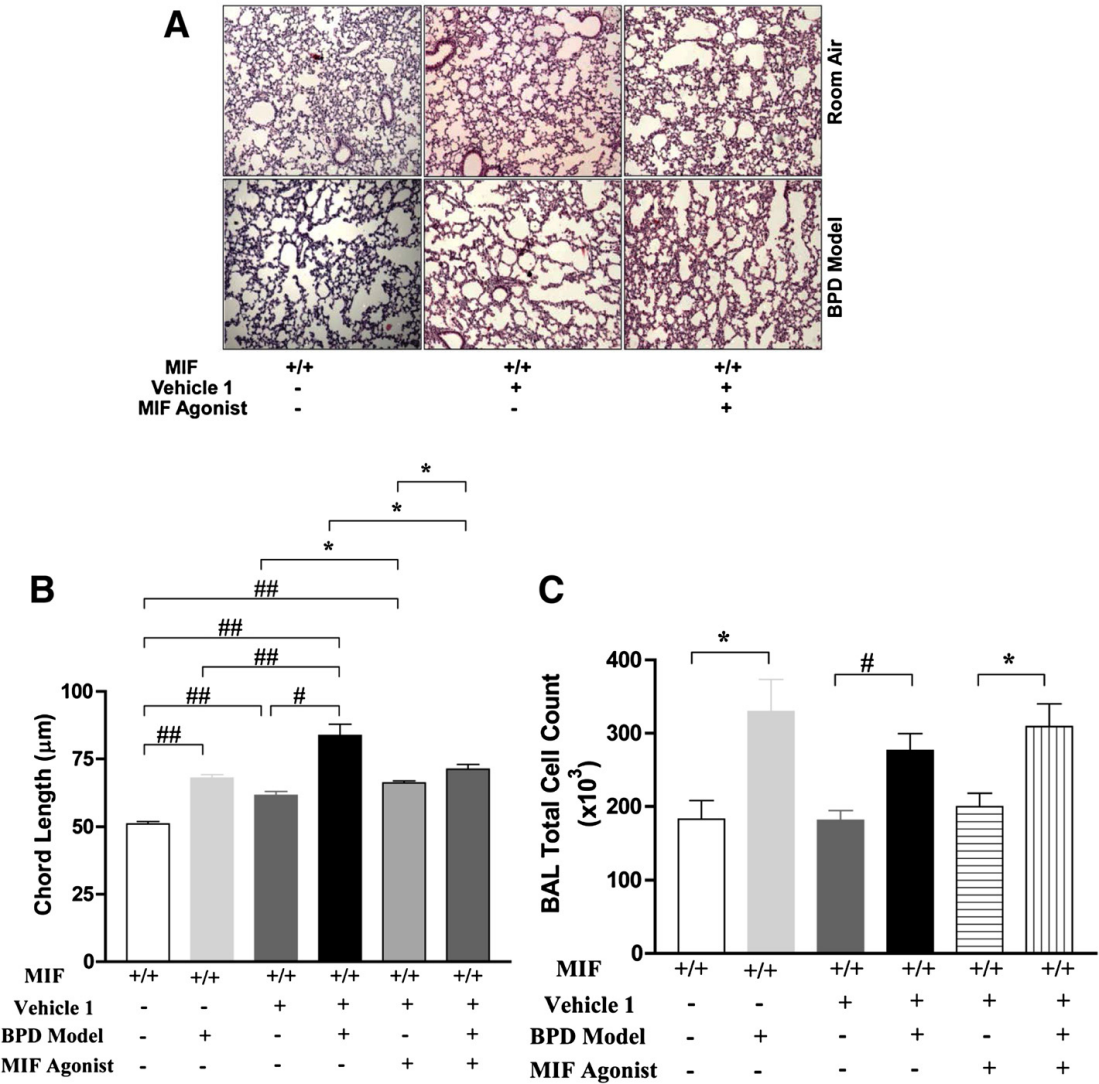
**Impact of MIF agonism in the mouse BPD model as reflected by pulmonary phenotype and BAL total cell counts.** NB WT control mice were exposed to 100% O_2_ for PN1-4, with recovery in RA from PN5-14 (BPD model). From PN1-14, some mice were treated with vehicle 1 or the MIF agonist, MIF020 (0.04 mg/g/d; dissolved in vehicle 1). Representative photomicrographs of lung histology (H&E stain) of NB WT mice exposed to RA or hyperoxia (BPD model), or given treatment as noted above, at PN14 (**5A**). The figures are illustrative of a minimum of 3 animals in each group. Alveolar size, as measured by chord length, confirmed features noted on lung histology (**5B**). Each bar represents the mean ± SEM of a minimum of three animals. BAL total cell count of NB WT mice exposed to RA or BPD model, or given treatment as noted above, at PN14 (**5C**). Each bar represents the mean ± SEM of a minimum of three animals. MIF +/+: wild type; MIF −/−: macrophage migration inhibitory factor knock out; BPD Model: bronchopulmonary dysplasia mouse model; Vehicle 1: 10% DMSO; MIF Agonist: MIF020; BAL: bronchoalveolar lavage. ##*P*<0.0001, #*P*≤0.01, *P<0.05.

Small molecule modulators of MIF signaling recently have been described that either enhance or inhibit MIF interaction with its receptor, CD74 [9,10]. Since lack of MIF predisposes to lung immaturity and the development of BPD, we tested whether the administration of the MIF agonist, MIF020, would ameliorate the pathologic effects of the BPD pulmonary phenotype. As shown in Figure 5A and B, MIF020 improved alveolar architecture, although the vehicle alone (10% DMSO in PBS) was associated with a modest increase in chord lengths. Enumeration of cells in BAL fluid also revealed increased values in all 3 BPD groups when compared to their respective RA controls (Figure 5C).

### Role of MIF antagonist in the mouse MIFTG-BPD model: impact on pulmonary phenotype and BAL total cell counts

**Figure 6:**
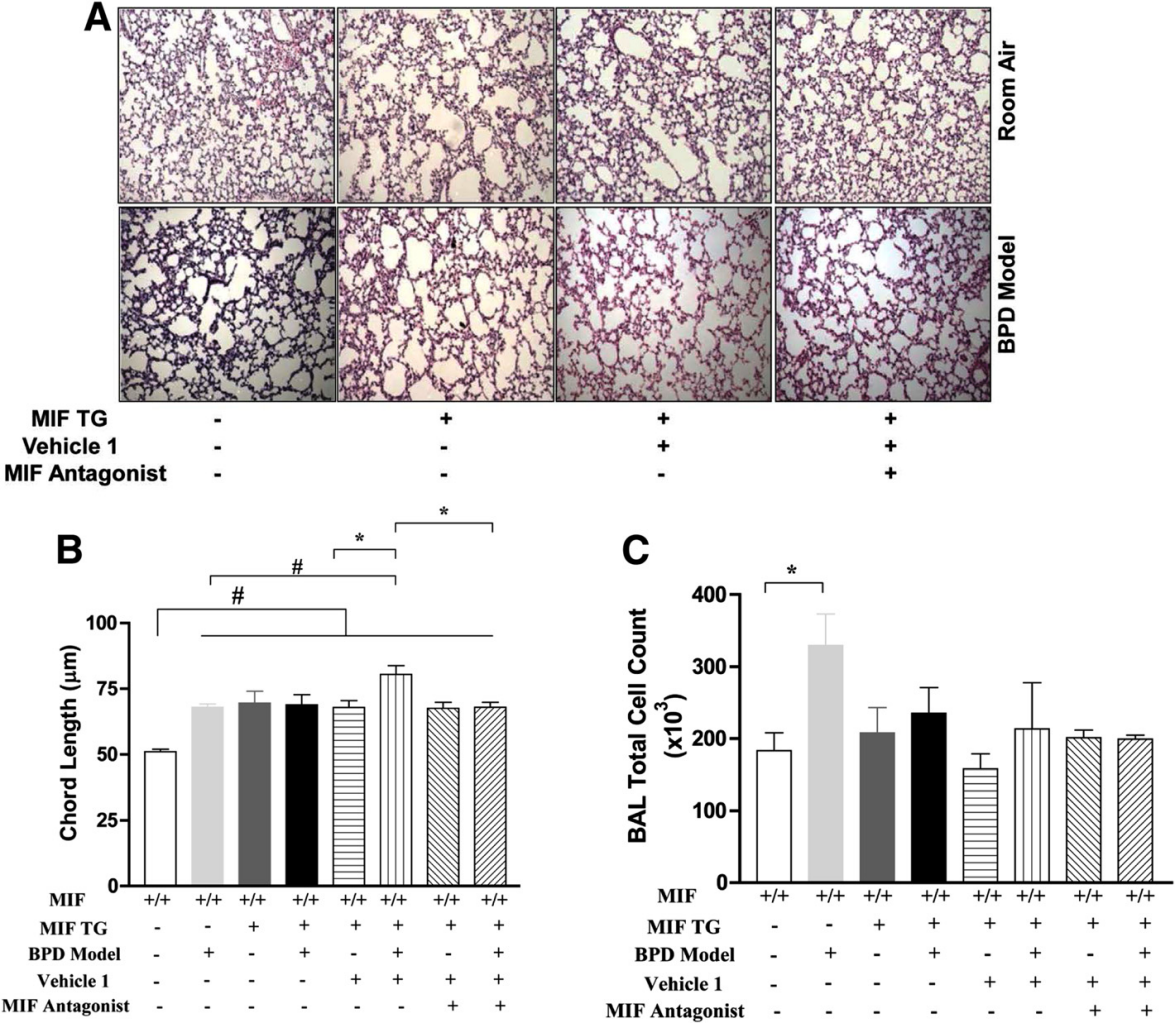
**Impact of pharmacologic MIF antagonism in the mouse MIF TG BPD model as reflected by pulmonary phenotype and BAL total cell counts.** NB MIF TG or WT mice were exposed to 100% O_2_ for PN1-4, with recovery in RA from PN5-14 (BPD model). From PN1-14, some mice were treated with vehicle 1 or the MIF antagonist, MIF098 (0.04 mg/g/d; dissolved in vehicle 1). Representative photomicrographs of lung histology (H&E stain) of NB MIF TG or WT mice exposed to RA or hyperoxia (BPD model), or given treatment as noted above, at PN14 (**6A**). The figures are illustrative of a minimum of 3 animals in each group. Alveolar size, as measured by chord length, confirmed features noted on lung histology (**6B**). Each bar represents the mean ± SEM of a minimum of three animals. BAL total cell count of NB MIF TG or WT mice exposed to RA or BPD model, or given treatment as noted above, at PN14 (**6C**). Each bar represents the mean ± SEM of a minimum of three animals. MIF TG +: macrophage migration inhibitory factor (over-expressing) transgenic; BPD Model: bronchopulmonary dysplasia mouse model; Vehicle 1: 10% DMSO; MIF Antagonist: MIF098; BAL: bronchoalveolar lavage. #*P*≤0.01, *P<0.05.

Since an excess of MIF also predisposes to BPD, we tested the effect of the MIF antagonist, MIF098 [10], in the MIFTG-RA and MIFTG-BPD models. The administration of MIF098 significantly improved alveolar architecture in the MIFTG-mice in the BPD model (Figure 6A and B). The observed improvement in alveolar architecture was partial at the dose tested however, and did not reach WT-RA values (Figure 6A and B). Total cell counts in BAL fluid revealed increased numbers in the WT-BPD group, compared to WT-RA control (Figure 6C), with no differences in total BAL cell counts in any of the other groups. Interestingly, BAL fluid cell counts were very similar in the MIFTG-BPD group treated with the MIF antagonist when compared to the similarly treated MIFTG-RA group (Figure 6C), and this observation parallels the similar chord length values recorded in these two experimental groups (Figure 6A and B).

These data suggest that MIF antagonism may influence alveolar architecture in the MIFTG-BPD lung by decreasing the ensuing inflammatory response. The partial improvement in alveolar architecture in the MIFTG-BPD model treated with MIF098 is probably due to the fact that the action of excess MIF occurs unabated after embryonic (E) day 14 (when the lung MIFTG is activated within the Type II alveolar epithelium) and the antagonist was administered only after PN1.

## Discussion

The present studies were undertaken to better define MIF’s role in the development of BPD, and employed both genetic loss- and gain-of-function strategies with MIFKO and MIFTG mice, respectively. We used the hyperoxia-induced BPD model and tested the efficacy of a recently developed, small molecule MIF agonist and antagonist.

Hyperoxia is a significant cause of lung injury and contributes to the pathogenesis of BPD [7] and our initial evaluation revealed a significant decrease in MIF expression at PN4 after hyperoxia exposure. In our mouse BPD model, at PN14, MIF expression was also markedly decreased compared to controls. These data are in accord with our previously reported significantly decreased MIF concentrations in tracheal aspirates in the first 48 h of NB human subjects (in the saccular stage of lung development) exposed to hyperoxia who go on to develop BPD at 36 weeks corrected postmenstrual age, compared to those who do not develop BPD [2]. Support for decreased levels of MIF being involved in the pathogenesis of BPD has also been demonstrated in tracheal aspirate fluid from extremely premature infants exposed to systemic fetal inflammation early in life [3]. Antenatal exposure to inflammation has been associated as one of the factors playing in the development of BPD [5,22,23].

In addition to impaired alveolarization, there is dysregulated vascularization in BPD [5]. The mouse model of BPD described previously by us [15] and others [20,24] shows long-term pathologic consequences similar to human BPD [25]. MIFKO and MIFTG mice in RA at PN14 showed altered alveolar architecture that did not worsen further upon hyperoxia exposure. This reiterates two points: first, an optimal amount of MIF is required for normal lung development, and second, the severity of impaired alveolarization with lack or excess of MIF is equivalent to that caused by hyperoxia in the WT developing lung in the saccular stage, with no additive effect of hyperoxia in the MIFKO and MIFTG mouse lung phenotype. We suggest that the need for “just the right amount” of MIF is important, and this (the “Goldilocks effect”) has been recognized by other investigators in relation to lung [26] and inner ear [27] development. This specific stage of lung development (i.e. saccular) being crucial for development of the murine BPD phenotype in relation to cytokine overexpression in other TG models has been previously noted by us [15] and others [28].

A dysregulation in vasculogenesis is another critical component of BPD. Hence, an evaluation of the developmental expression of vascular mediators in these models has the potential to provide mechanistic insight. Ang1 protein expression was decreased in the WT-BPD, MIFKO-BPD and MIFTG-BPD, while Ang2 was increased in all 3 BPD groups (Figure 4). Our data would suggest an important role of the Ang2-Ang1 ratio in the context of MIF signaling in the BPD model. This has potential translational significance given the reported association of increased Ang2 [19,29] and decreased Ang1 [30] with human BPD.

The administration of a pharmacologic MIF agonist that enhances MIF binding to its receptor was found to be partially protective in the WT-BPD model. We did note some adverse impact on alveolarization of the vehicle used (10% DMSO) to dissolve the agonist. We used the dose noted in studies conducted with adult mice [31], which probably led to the adverse impact on alveolarization. A lower concentration of DMSO would be preferred for future studies, as DMSO has been used safely in hyperoxia-exposed neonatal mice [32,33]. Conversely, the administration of a small molecule MIF antagonist that blocks MIF binding with its receptor also was protective in the MIFTG-RA and MIFTG-BPD models. Taken together, these data suggest that restoring MIF activity within the lung to “just the right amount” is beneficial to the development of normal alveolar architecture.

These data suggest that the mechanism of hyperoxia-induced BPD in the murine model may be similar to lack of MIF. This notion is made further plausible by recent observations that MIF expression is strongly governed by the activation of hypoxia-inducible factor 1α (HIF-1α) [34,35]. Lack of HIF has been associated with animal models of BPD [36,37], and this observation provides a potential mechanistic link. Furthermore, increased Ang2 has been associated with decreased HIF-1α [38]. This notion underscores prior observations that both a lack and an excess of angiogenesis during a critical stage of pulmonary development disrupts alveolarization [14,39-41]. While a considerable amount of data has been published on the lack of vascular development as a hallmark of BPD [39,42], we posit that an excess of pro-angiogenic factors [14,17,19,40,42] during the critical stage of lung development also lead to the same end result. We suggest that further investigations into the role of decreased MIF activity leading to decreased HIF-1α action, and the association with increased Ang2-decreased Ang1-Tie2 signaling pathway, would be fruitful in understanding the pathogenesis of BPD. Since the functioning of these molecules are context-dependent, studies need to be pursued in developmentally-appropriate pulmonary models.

Recently, the MIF −173*C allele, which predisposes to higher MIF production, was associated with a decreased incidence of BPD, independently from mechanical ventilation and oxygen exposure [4]. Conceivably, use of small molecule MIF modulators as a therapeutic approach to prevent BPD may be guided by a patient’s genetic predisposition based on *MIF* alleles.

## Conclusions

In summary, we noted that a lack or excess of MIF in the developing lung both lead to an alveolar simplification pulmonary phenotype, which was not further worsened by hyperoxia exposure in the survivors. These effects were associated with alterations in the protein expression of the Ang1, Ang2 and Tie2 angiogenic factors. Use of a MIF agonist in the WT-BPD or MIF antagonist in the MIFTG-BPD models restored the pulmonary phenotype towards normalcy. We speculate that the Ang1-Ang2-Tie2 axis signaling pathway mediates the pulmonary effects of MIF in the developing lung.

The present findings have clinical relevance and support our earlier work showing lower MIF tracheal aspirate levels in human infants developing BPD [2]. We speculate that restoring or enhancing MIF activity in the developing lung, without reaching supra-physiological levels, has the potential to improve impaired alveolarization in the infants at risk for BPD. Additional research is needed to ascertain the ideal circumstances for augmenting MIF action in the lung, potentially via the systemic or intra-pulmonary application of small molecule MIF modulators that would be protective of the developing lung predisposed to BPD.

## Competing interests

Yale University has applied for a patent describing the therapeutic utility of MIF agonists in neonatal lung development.

## Authors’ contributions

Concept and Design: HS, WJ, RB, VB. Acquisition of data: HS, R C-W, JF, LL, MS, AH. Data analysis and Interpretation: HS, WJ, RB, VB. Drafting and/or Critical revision for intellectual content: HS, R C-W, JF, LL, MS, AH, WJ, RB, VB. All authors have approved the version of the submitted manuscript.
